# Target Design in SEM-Based Nano-CT and Its Influence on X-ray Imaging

**DOI:** 10.3390/jimaging9080157

**Published:** 2023-08-04

**Authors:** Jonas Fell, Felix Wetzler, Michael Maisl, Hans-Georg Herrmann

**Affiliations:** 1 Lightweight Systems, Department of Materials Science, Campus E3 1, Saarland University, 66123 Saarbrücken, Germany; felix.wetzler@hs-kl.de (F.W.); hans-georg.herrmann@izfp.fraunhofer.de (H.-G.H.); 2 Fraunhofer Institute for Nondestructive Testing IZFP, Campus E3 1, 66123 Saarbrücken, Germany; michael.maisl@izfp.fraunhofer.de

**Keywords:** SEM-based CT, X-ray target, X-ray source, image quality, nano-CT, spatial resolution

## Abstract

Nano-computed tomography (nano-CT) based on scanning electron microscopy (SEM) is utilized for multimodal material characterization in one instrument. Since SEM-based CT uses geometrical magnification, X-ray targets can be adapted without any further changes to the system. This allows for designing targets with varying geometry and chemical composition to influence the X-ray focal spot, intensity and energy distribution with the aim to enhance the image quality. In this paper, three different target geometries with a varying volume are presented: bulk, foil and needle target. Based on the analyzed electron beam properties and X-ray beam path, the influence of the different target designs on X-ray imaging is investigated. With the obtained information, three targets for different applications are recommended. A platinum (Pt) bulk target tilted by 25° as an optimal combination of high photon flux and spatial resolution is used for fast CT scans and the investigation of high-absorbing or large sample volumes. To image low-absorbing materials, e.g., polymers or organic materials, a target material with a characteristic line energy right above the detector energy threshold is recommended. In the case of the observed system, we used a 30° tilted chromium (Cr) target, leading to a higher image contrast. To reach a maximum spatial resolution of about 100 nm, we recommend a tungsten (W) needle target with a tip diameter of about 100 nm.

## 1. Introduction

Compared to electron imaging, in SEM-based nano-CT, the electron beam is focused on an X-ray target, leading to photon emission. In combination with a suitable X-ray detector, X-ray imaging can be realized by direct magnification. The method takes advantage of a fine electron spot size, leading to the formation of a small X-ray focal spot, resulting in a spatial resolution down to about 100 nm for X-ray imaging [1,2]. Therefore, the system expands the modalities of SEM and is highly suitable for correlative microscopy [2,3,4,5].

Since SEM-based CT utilizes geometrical magnification for imaging, the hardware only consists of an X-ray target, a rotary sample stage available from SEM and an X-ray detector. The setup does not use X-ray optics tuned to a specific X-ray energy. In contrast, the X-ray target is easily accessible so it can be exchanged and adapted to the requirements of the measurement and no further changes to the system are needed. This allows for a variety of X-ray target modifications in terms of chemical composition, geometry and tilt. Varying these parameters allows for influencing X-ray beam characteristics like the energy distribution, intensity and focal spot size, which affects image quality parameters like the contrast and spatial resolution. In the past, different materials and shapes have been used, but a detailed correlation between the target design and its influence on X-ray imaging has not been investigated in detail. Research has been focused on improving the spatial resolution by minimizing the interaction volume of the electron and target and therefore reducing the target size. The target shape varies from bulk material [6], thin foils [7,8,9], to small wedge-shaped geometry, down to wire-shaped [10] and needle-shaped targets with a tip diameter of about 100 nm [3]. Target materials like aluminum, titanium, tungsten, tantalum, gold or platinum–iridium alloys have also been mentioned but very few of them have been characterized [7,11]. In addition, only the melting point has been taken into account, especially if the size of the target is very small.

We focus on an overall view of the target design, electron–matter interaction and its influence on X-ray beam properties with the goal to enhance the X-ray image quality. In the first step, the electron beam properties are determined as a base of the X-ray source. With knowledge about the electron beam and with the support of simulations, different targets with a varying geometry and chemical composition are analyzed experimentally in the second step. As a result, we present different target designs, leading to different X-ray beam properties suitable for various applications.

## 2. Materials and Methods

### 2.1. Experimental Setup

**Figure 1:**
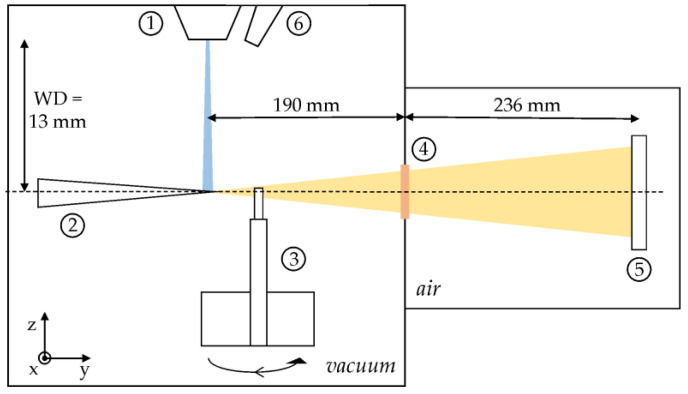
Illustration of XRM-II nano-CT: The electron beam (blue) generated in the electron column (1) interacts with an X-ray target (here: needle-shaped target), (2) leading to X-ray emission (orange) used for imaging. X-rays are attenuated by the specimen mounted to a sample holder (3) and have to pass a Be window (4) and travel through air before hitting the X-ray detector (5). An EDS detector allows for chemical analysis for material characterization (6).

XRM-II nano-CT (ProCon X-ray, Sarstedt, Germany) is a system based on the field emission SEM JEOL JSM-7900F (JEOL, Tokyo, Japan) and illustrated in Figure 1. In addition to a secondary electron detector, the system is also equipped with an EDAX Element system (EDAX–AMETEK, Berwyn, PA, USA) for energy dispersive X-ray spectroscopy (EDS). Inside the electron column, the instrument is equipped with a probe current detector (PCD) to measure the electron beam current after passing through the optical system and aperture. There is a set of different aperture diameters that can be exchanged to vary the electron beam spot size and electron current. In addition to the PCD, a picoampere meter is integrated into the sample stage to measure the absorbed current of the sample (ACM). With an X-ray target manipulator inside the vacuum chamber and a photon counting X-ray detector (PCXD) (WidePIX from ADVACAM, Praha, Czech Republic) attached to it outside the vacuum chamber, a cone beam CT based on geometric magnification can be realized. The PCXD consists of 2 × 5 Medipix3 devices with a pixel size of 55 µm, leading to a total amount of 1280 × 512 pixels. The detector is constructed with a 1 mm CdTe sensor layer.

For nano-CT, the electron beam is focused on a target emitting a continuous X-ray spectrum, due to Bremsstrahlung and characteristic X-ray emission, which is attenuated by the sample volume right in front of the target. X-ray attenuation is determined by the chemical composition and density of the investigated sample. After penetrating the object, photons leave the vacuum chamber by passing through a 250 µm Beryllium (Be) window and hit the PCXD at a distance of about 426 mm to the X-ray source. Due to an energy threshold, the PCXD detects photons only above 5 keV. Further description of the CT mode of the XRM-II as well as volume reconstruction can be found in [2,3,5]. The aforementioned equipment of the PCD, ACM, PCXD, EDX detector and electron imaging is used for experimental characterization.

### 2.2. Simulation of Electron–Target Interaction

In addition to experimental characterization, simulations are applied to evaluate the parameters of the X-ray source, which are not accessible via experiments. For this purpose, we used the simulation software CASINO (v2.48, Université de Sherbrooke, Québec, Canada) [12] and NIST DTSA-II (Lorentz 2020-05-18 revision, National Institute of Standards and Technology, Gaithersburg, MD, USA) [13]. Both software are based on Monte Carlo simulation and provide a different scope of simulation tasks. CASINO is specially designed to simulate the electron trajectories of low-energy beam interaction in an SEM and is used to determine parameters like the backscatter coefficient or electron penetration depth. DTSA-II allows for the simulation of X-ray spectra as a result of the interaction with materials of a different geometry and chemical composition. Since DTSA is designed to simulate EDS, only Si(Li) and SDD detectors are available in the software, leading to a discrepancy in absorption efficiency compared to CdTe as the sensor material in the XRM-II. To reach a photon absorption of nearly 100%, like with CdTe, the sensor thickness is enhanced to a maximum of 100 mm. In both software, the setup of the components and electron beam parameters are adapted to the XRM-II.

## 3. Results and Discussion

### 3.1. How to Influence X-ray Beam Properties in SEM-Based Nano-CT

Multiple parameters influence the properties of X-ray radiation in SEM-based CT. The two most important parameters are the electron beam properties and X-ray target properties. The following sections outline a detailed characterization of the electron beam and X-ray target as well as the consequences of their interaction for X-ray imaging.

#### 3.1.1. Electron Beam Characteristics

A precise knowledge of electron beam characteristics is necessary to quantify the electron–matter interaction of the X-ray source and as the input for electron simulations. Electron beam properties are mainly determined by the electron energy, electron current and electron spot size, which are investigated in the following.

The electron energy has a major influence on the emitted X-ray spectrum and X-ray focal spot. In SEM, the electron energy can reach 5–30 keV by selecting the corresponding acceleration voltage so the X-ray photon energy is limited to a maximum of 30 keV. The choice of acceleration voltage also determines the characteristic X-ray excitation, which should be about two to three times higher for an optimal photon outcome [14]. This is important because characteristic X-ray photons contribute strongly to the X-ray intensity of the spectrum. Furthermore, the electron energy determines the mean free path and penetration depth of electrons in matter and leads to a specific extension of the electron–matter interaction volume. Its size correlates with the X-ray focal spot size and influences the spatial resolution in X-ray imaging, which is discussed later on. The electron beam spot size is another important parameter, which also slightly influences the electron energy, as explained later on.

The electron beam current is the second parameter to look at and strongly correlates with the used electron aperture. There are different apertures at two different positions installed in the electron beam path. One is an aperture at the top of the electron column with a diameter of 2 mm, which reduces misaligned electrons and is called the noise canceller (NC). The second one is the objective lens aperture (OLAP), positioned at the lower end of the column, which is a set of four apertures with different diameters between 30 and 1000 µm, as shown in Table 1.

The electron beam current can be influenced in two ways: Firstly, by manually exchanging the aperture in the electron column, which limits the electron spot size and therefore results in a change in the electron beam current. Secondly, by incrementally changing the beam current controlled in the software (PC values), which causes a variation in the electron spot size as well. In terms of X-ray imaging, a high electron current is of interest to reach the maximum X-ray intensity.

**Figure 2:**
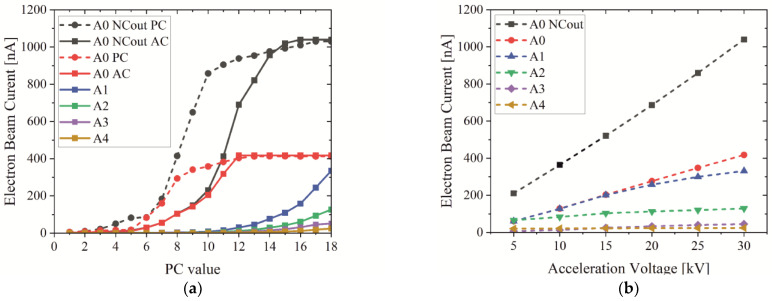
(**a**) A 30 keV electron beam current depending on PC value for different apertures; (**b**) electron beam current depending on acceleration voltage for different apertures (PC 18).

Depending on the selected apertures, the electron beam current is measured with the PCD in the electron column and validated with a Faraday cup and ACM on the sample stage. The results are shown in Figure 2a for different probe current values, which can be selected in the software. The electron probe current (PC) measured with PCD perfectly matches the absorbed current (AC) measured with the Faraday cup except for the use of A0. For conventional SEM usage, this aperture is not used for imaging and only for beam alignment. The measured effect can be explained by the beam divergence and scattering of misaligned electrons. Despite the measured discrepancy, this fact can be neglected because only the maximum current (PC 18) is relevant for X-ray imaging and in this case, the PC and AC are identical. A1 results in a maximum electron beam current of about 330 nA. Smaller apertures are not useful for X-ray imaging and result in a very low X-ray intensity and extremely long image acquisition time. In the case of A0, the spot size increases and a maximum current of about 440 nA can be reached, which enhances to about 1 µA without the NC. Furthermore, the selected acceleration voltage influences the electron beam current, as shown in Figure 2b, for different apertures. For a high X-ray flux, only an acceleration voltage of 30 keV is most effective. Another advantage of a higher electron energy is the increasing probability of characteristic X-ray excitation. The only disadvantage of high-energy electrons is an increased interaction volume and X-ray focal spot. Nevertheless, the latter can also be minimized by the target geometry, as explained in Section 3.2.

**Figure 3:**
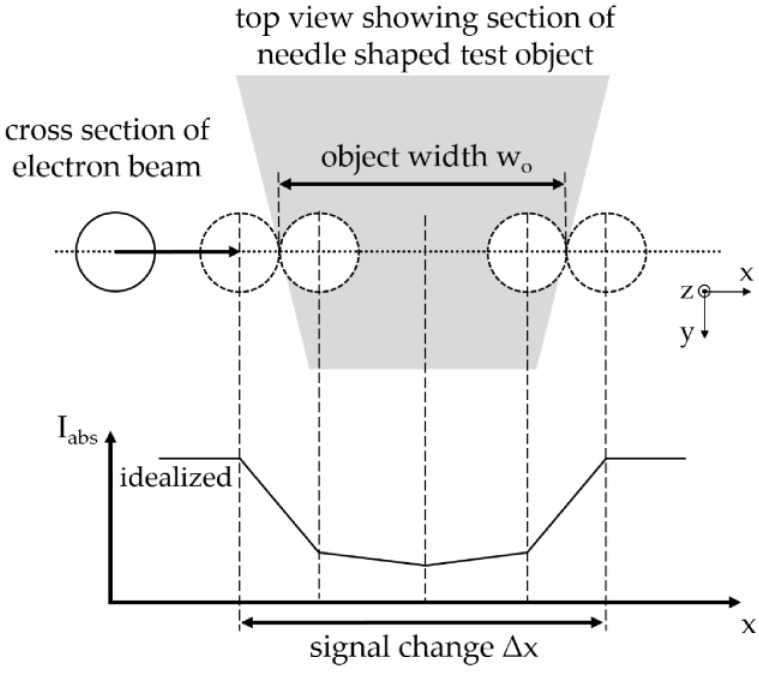
Principle for measuring electron beam diameter: Scanning over an object (here: needle) leads to signal change in absorbed current. Beam diameter can be calculated with the known object width.

Another essential parameter of SEM-based CT is the electron spot size, which determines, in combination with the electron current, the number of electrons per area hitting the target or the electron density, respectively. Estimating the electron spot size with the help of the spatial image resolution, as is common in SEM, is not suitable because of high electron currents, leading to thermal drift or the melting of the test objects. To determine the electron spot size, a modified method from Zhao et al. [15] was adapted to the provided hardware. As shown in Figure 3, a needle made of tungsten is used as the test object and placed on a brass sample holder, which is connected to an ACM. While the spot mode is activated, the AC is measured by moving the electron beam stepwise along a line over the W needle. The test object size (here: needle diameter) should be significantly larger than the electron beam step size to obtain a clear measuring signal. Moreover, the object should not be too large since a longer measurement time leads to thermal drift of the system and inaccurate measurements. A minimum object size is not given. In the presented case, we used a needle with a diameter of about 400 nm and an electron beam step size of 10–20 nm for measuring. As soon as parts of the electron beam hit the object surface, the AC reduces tremendously due to the high backscatter coefficient of W. As soon as the entire electron beam hits the target, the absorbed current reduces only slightly as long as electrons transmit the W target. The electron spot size s_e_ can be calculated by subtracting the object width w_o_ from the distance of the signal change Δx:

s_e_ = Δx − w_o_. (1)

Since only the position of the signal change is used to calculate the beam spot size, the method is independent of the object shape. Moreover, the result delivers the maximum extension of the electron beam regardless of the electron density distribution or Gaussian distribution, respectively. Therefore, it will be an overestimation of the effective electron spot size.

**Figure 4:**
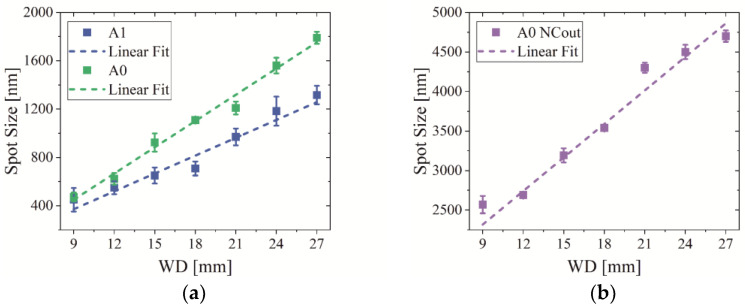
(**a**) Electron spot size depending on WD for apertures A1 and A2; (**b**) spot size depending on WD for apertures A0 with removed NC. (Measuring parameter: 30 kV, PC 18).

Different parameters like the aperture, PC value, electron energy and working distance (distance between the electron exit point and focus plane (WD)) influence the electron spot size. As discussed before, suitable parameters for X-ray imaging are PC 18, aperture A1 or A0 and an electron beam energy of 30 keV. The latter is also the best choice because a higher electron energy leads to a smaller electron spot size due to a lower beam divergence. The mentioned electron beam parameters are selected to measure the electron beam spot size with the developed method depending on the WD. The results are depicted in Figure 4 and show a strong change in the electron spot size. Reducing the WD by about 10 mm minimizes the electron beam spot by about one-half for A0 and A1. In case the NC is removed from the beam path, the overall spot size is strongly increased but it can also be reduced by lowering the WD. The optical axis for X-ray imaging with the XRM-II is adjusted to a WD of 18 mm. Due to the presented data, the setup should be modified to obtain the smallest possible WD to reach the maximum efficiency.

Due to the exit window flange blocking the X-ray beam path, the optical axis is geometrically limited to a WD of 13 mm so the PCXD is only lifted up by about 5 mm. Because of the modification, a smaller electron beam spot and higher electron density are achieved so the X-ray intensity is enhanced in the case of a needle target and the X-ray focal spot size is minimized in the case of a bulk or foil target.

To complete all the influences on the electron beam properties, the operator has to be mentioned as a parameter. Alignment of the focus and astigmatism is operator-dependent and has a significant influence on the electron beam shape and spot size hitting the target surface.

#### 3.1.2. Geometry of X-ray Targets

In addition to the electron beam characterization, we investigated different target designs with a varying chemical composition, geometry and tilt angle. As previously discussed, a limitation of the X-ray focal spot size can be realized by reducing the acceleration voltage. A lower electron energy leads to a lower electron penetration depth and better spatial resolution for X-ray imaging will be achieved. At the same time, this would lead to disadvantages like a larger electron spot size (Figure 4a) due to the electron beam divergence and lower emission probability of characteristic X-rays. A more effective method is limiting the geometrical extension of the target. The target geometry and tilt determine the size of the electron–matter interaction volume, influencing the emitted X-ray intensity and spatial resolution in X-ray imaging.

Due to the dimension of an X-ray target, we distinguished between the following geometries:

- Bulk target: The target size is significantly larger than the interaction volume of the electrons and matter. No electrons will be transmitted through the target.
- Foil target: A thin target layer below the size of the electron–matter interaction volume in one dimension. Typical dimensions are below 1 µm.
- Needle target: The target geometry is similar to a needle and the interaction volume is strongly limited in two dimensions. The needle tip is pointing toward the X-ray detector.

**Figure 5:**
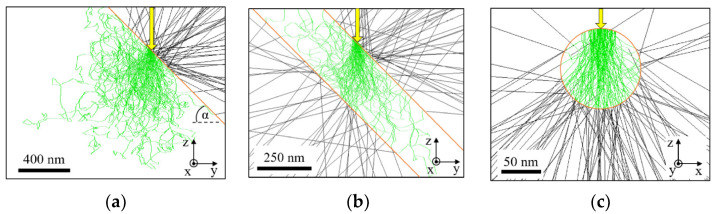
Cross-section of electron–target interaction volume: (**a**) bulk target; (**b**) 250 nm foil target; (**c**) needle target with 100 nm in diameter. Simulation is performed with DTSA with W defined as target material and an incoming electron beam size of 50 nm under an angle of 45°. α is defined as target tilt angle. Yellow arrows indicate incoming electron beam and object surface is drawn in orange. Green electron trajectories represent the traveled distance within the target and black trajectories represent backscattered or transmitted electrons. Bulk and foil targets are depicted in side view and needle target in front view.

The different target geometries are visualized in Figure 5, where incoming electrons are depicted in yellow and electron trajectories within the target material in green. Backscattered and transmitted electrons are represented in black.

Regarding the bulk target, electrons reach the maximum penetration depth and the X-ray focal spot extends to its full size. In this case, the electron–target interaction volume (more details in Section 3.3.1), which is equivalent to the X-ray focal spot, approximately determines the spatial resolution in X-ray imaging. As soon as the target tilt and detector position are taken into account, the effective X-ray focal spot size, defined as the projection of the X-ray focal spot onto the detector surface, is the relevant parameter for spatial resolution. The spatial resolution in the x-direction is independent of the target tilt angle and determined by a combination of the electron spot size and lateral electron propagation. The spatial resolution in the z-direction has to be distinguished depending on the target tilt angle. For low target tilt angles, the electron penetration depth determines the spatial resolution while for high target tilt angles, the electron spot size determines the spatial resolution. In terms of the X-ray intensity, the bulk target reaches a maximum since incoming electrons transfer their complete energy into the target.

Foil (Figure 5b) or needle targets (Figure 5c) show different behaviors, which limit the spatial extent of electrons so only part of the electron energy is used for ionization and X-ray emission, before transmitting the target. Consequently, the focal spot size is reduced, the spatial resolution is enhanced but the X-ray intensity is decreased. In the case of a foil target, the X-ray focal spot size is determined by the same parameters as explained for the bulk target with one exception: for low target tilt angles, the electron penetration depth is limited by the foil thickness so the spatial resolution in the z-direction is also determined by the foil thickness. In the case of a needle target, the target geometry determines the X-ray focal spot size in the x- and z-direction. Another effect of the geometrical limitation of the X-ray focal spot size is an increase in the X-ray energy. Since high-energy photons are only emitted near the target surface, the energy of the emitted photon decreases with the increasing layer thickness and electron penetration depth, respectively. For example, reducing the layer thickness of a W foil target from 500 nm to 100 nm increases the mean X-ray photon energy by 1 keV, as simulations show.

Reducing the WD has no influence on the X-ray intensity while using a bulk or needle target, since the number of electrons hitting the target does not change. This is different for the use of a needle target. A smaller electron spot size at a low WD leads to an increased electron density hitting the target and therefore the X-ray intensity increases. For bulk and foil targets, the X-ray intensity can be influenced by the target tilt and will be discussed in detail in Section 3.3.1. Further investigations of the needle target are presented in Section 3.3.2.

#### 3.1.3. Chemical Composition of X-ray Targets

The choice of target material determines the energy distribution of the emitted X-ray spectra, influences the behavior of attenuation and thereby the contrast of images. The characteristic X-ray line energy is highly important to select a suitable target material because characteristic photons strongly contribute to the X-ray spectrum. As listed in Table 2, the amount of characteristic photons varies from about 10–80% regarding the entire X-ray spectrum in the range of 5–30 keV and leads to a large difference in the overall X-ray intensity. The intensity of characteristic X-rays depends on their energy in combination with the excitation energy. As mentioned before, the excitation energy needs to be at least two to three times higher than the characteristic X-ray line energy to achieve the maximum intensity. Since the maximum electron energy in SEM is limited to 30 keV, the characteristic X-ray energy should not exceed an energy of around 15 keV for a high intensity. In addition, the detected X-ray energy is limited to 5 keV due to the PCXD’s energy threshold. Further important parameters of the target material are the melting point and thermal conductivity, which determine the thermal stability, especially for small targets. Based on these facts, Table 2 shows potential target materials with different characteristic X-ray line energies in a range of around 5–15 keV, their melting point and their relevant characteristic X-ray line energy. Additionally, a maximum emission depth of a photon with an energy of 5 keV is listed. Elements with low melting points and thermal conductivity are only suitable for foil or bulk target geometry.

Due to the high thermal energy input of the focused electron beam, parameters like the melting point and thermal conductivity are important regarding the target geometry. In contrast to a bulk target, which can be realized with every material, a needle target can only be realized by using materials with a very high melting point like W or in combination with a low electron flux. To take advantage of a smaller target volume, manufacturing a needle target embedded into a diamond substrate or a foil target sputtered onto a diamond substrate could also be realized. Thus, the spatial resolution for materials with a high electron penetration depth like Cr or Y will be enhanced.

The selected target material also influences the X-ray focal spot size especially in the case of a bulk material since the interaction volume is not limited by the target geometry. The incoming electron energy E_0_ (in keV), atomic number Z, atomic mass number A (in u) and density ρ (in g/cm^3^) of the target material determine the probability of the electron–matter interaction and therefore the electron penetration depth. In addition, only electrons with an energy above E_c_ = 5 keV can excite photons used for imaging due to the PCXD’s detector threshold. Castaing or Gaber and Fitting define the maximum X-ray emission depth z_m_ (in nm) for incoming electrons perpendicular to the object surface as [16,17]:

d = (0.033 (E_0_^1.7^ − E_c_^1.7^) A)/(ρZ), (2)

Based on the approximation, a maximum X-ray emission depth of a 5 keV photon is calculated for potential target materials and listed in Table 2. As an example, W or Pt strongly limit the electron penetration, leading to a maximum photon emission depth of about 1 µm, while the maximum photon emission depth of light metals like Cr is about three times higher. In the case of the XRM-II, one has to keep in mind that the approximation will be an overestimation due to different facts. First, X-ray imaging is determined by a volume of high photon density and not by photons with the lowest detectable energy. Second, the target is tilted for imaging, leading to a lower electron penetration depth and therefore a reduction in the X-ray focal spot size. Third, only the effective X-ray focal spot size (projection of the X-ray focal spot onto the detector surface) is relevant for imaging.

A further parameter influencing X-ray radiation is the X-ray beam path determined by the setup, which will be discussed in the following. Later on, we present the characterization of different target geometries and materials in terms of the X-ray intensity and spatial resolution in X-ray imaging.

### 3.2. X-ray Beam Path in SEM-Based CT

In X-ray imaging, the emitted and detected X-ray spectra vary because of the detection efficiency of the detector. In the case of the XRM-II, the beam path leads to additional photon absorption and is quantified in the following.

**Figure 6:**
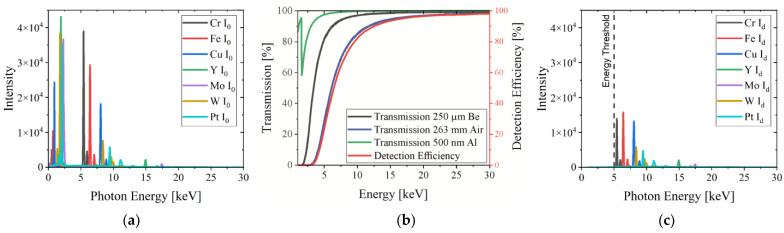
(**a**) Simulated X-ray spectra excited with 30 keV electrons for various target materials showing different characteristic X-ray line energies and massive variations in X-ray intensity; (**b**) detection efficiency depending on photon energy: transmission of X-ray photons passing through 250 µm Be, 236 mm air and 500 nm Al before interacting with the sensor material. Absorption efficiency of the sensor material made of 1000 µm CdTe is nearly 100% for the entire energy range; (**c**) detected X-ray spectra calculated with Lambert–Beer law showing absorption due to the beam path in XRM-II.

To calculate the emitted X-ray spectra for different target materials, the simulation software DTSA-II was used and the simulation parameters were adapted to the setup of the XRM-II (Monte Carlo simulation of a bulk; no detector window; maximum Si sensor thickness of 100 mm to reach 100% absorption; 0° detector tilt angle; 45° object tilt angle). The simulation results are represented in Figure 6a and show massive variations in the characteristic X-ray intensity. Materials with low characteristic line energies (Cr, Fe, Cu) show a high X-ray intensity since a large amount of electrons, even after multiple scatter events, are able to excite photon emission. Materials with high characteristic X-ray line energies (Y, Mo, W, Pt) show a low X-ray intensity because of a low probability of ionization and X-ray emission.

Since the X-ray detector of the XRM-II is placed outside the vacuum chamber, a Be window of 250 µm, transparent for hard X-rays, is necessary to separate the vacuum chamber from its environment. In addition to passing through the Be window, X-ray photons need to travel about 236 mm through air and pass a 500 nm Al layer in front of the sensor material before detection. Since the X-ray energy used in SEM-based CT is rather low, the absorption of these three components should be taken into account when calculating the X-ray intensity. The values of the total attenuation are generated from XCOM (NIST) at standard atmospheric pressure (1013 hPa) while air is defined as a mixture of 78% N, 21% O and 1% Ar [18]. Figure 6b shows the calculated transmission depending on the photon energy. The absorption efficiency of the sensor material made of 1 mm CdTe is nearly 100% for the relevant energy range and can be neglected in the examination. The overall effect of the three materials on the photon transmission is summed up in the detection efficiency and mainly influenced by air.

Using the detection efficiency and employing the Lambert–Beer law, detected photons attenuated by the beam path can be calculated and are represented in Figure 6c. Since the X-ray detector energy threshold cuts off photons below 5 keV, the relevant energy range is 5–30 keV. It can be clearly seen that the X-ray beam path in air reduces the photon flux by 50% in the energy range between 5 and 10 keV.

A quantitative evaluation is listed in Table 3, where the intensity (sum of the photons of the X-ray spectrum) of the emitted and detected photons as well as the resulting total transmission (difference between the emitted and detected photons) can be found. In addition, the median of the X-ray energy is listed. Regarding the absolute emitted intensity of different target materials, Cr is by far the highest one. Nevertheless, the detected intensity is quite low because low-energy photons and especially the characteristic peak at 5.41 keV are strongly absorbed in the X-ray beam path. In total, about 40% of the photons in the range of 5–30 keV are detected. Compared to that, the absorption of high-energy photons is significantly lower, but the probability to excite them is rather low. Therefore, the transmission of Y, Mo, W and Pt X-ray spectra is about 75–80%. The target materials W and Pt obtain the overall highest intensity due to a relatively high X-ray emission and transmission.

Transferring the beam path to a vacuum increases the X-ray intensity by about 20–50% depending on the target material. Consequently, the acquisition time for X-ray CT would decrease by about the same proportion. Unfortunately, the currently equipped PCXD is not suitable to be used in an ultra-high vacuum but adaptations to the system are possible. Similar results could be reached by flooding this part of the beam path with He, which also reduced the X-ray absorption tremendously.

To increase the contrast in X-ray imaging, the energy distribution of the X-ray spectra plays a decisive role. An indicator to select the right target material that fits the needs of an investigated specimen is the median of the detected X-ray energy (5–30 keV) shown in Table 3. Due to the beam path, the detected X-ray energy is enhanced and reaches values between 5.5 and 13.4 keV for the selected target materials. This information is used to influence the image contrast, as discussed later.

The beam path can also influence the spatial resolution in X-ray imaging since air scattering can broaden the beam and thus worsen the resolution. This effect is especially strong at low photon energies.

### 3.3. Target Design

Based on the presented results, a set of targets are designed to fulfill different tasks: one with a high X-ray intensity for fast measuring, another for a high spatial resolution and a third to enhance the image contrast for an investigation of low-absorbing materials.

#### 3.3.1. Bulk Target

To reach the maximum X-ray intensity, the obvious choice of geometry is a bulk target since the entire electron energy is used for X-ray emission. As shown in Table 3, the target should be made out of W or Pt due to a high detected X-ray intensity. An important parameter for the X-ray intensity and formation of the X-ray focal spot in a bulk target is the tilt angle, which is investigated experimentally.

**Figure 7:**
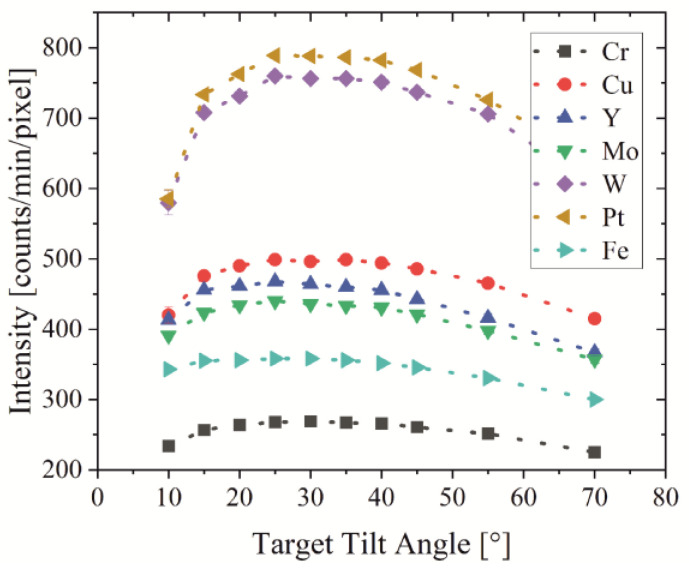
Experimental measurement of the X-ray intensity depending on target tilt angle for various materials.

In Figure 7, the experimental measurements of the X-ray intensity show a maximum at around a 25–30° target tilt angle for all targets, whereas Pt leads to the highest measured intensity of 800 photons per minute (330 nA electron beam current) followed by W. Additional measurements show that the use of aperture A0 and removal of the NC lead to an intensity of around 2300 counts/min for a 25° tilted Pt bulk target which is an increase of a factor of three.

The target materials Cu, Y and Mo reach about 60%, Fe about 50% and Cr about 40% intensity compared to Pt. These materials lead to a longer measurement time for a similar SNR, but they are important due to a different photon energy distribution, which results in differences in the image contrast. The relative simulated intensity listed in Table 3 deviates from the measured intensity: materials with characteristic photon energy photons close to the detector threshold, especially Cr, Fe and Cu, show a lower intensity in experiments compared to simulations. This originates most likely from the detector threshold being set to 5 keV. While standard values are about 8–10 keV, the selected value of 5 keV seems to be unstable and and detector threshold shifts to higher energies. Thermal drift may also lead to a higher energy threshold.

To understand the behavior of the X-ray intensity depending on the target tilt angle, further investigations were performed via DTSA simulations. Within DTSA, the target and detector position as well as detector pixel size can be adapted according to the XRM-II. The X-ray intensity is calculated as a sum of the X-ray spectrum depending on the target tilt angle, which is changed in increments of 5° between an absolute value of 5°and 85°. The intensity is evaluated for three different conditions: (i) emitted X-ray spectrum (energy range from 0–30 keV), (ii) emitted X-ray spectrum in the detectable energy range from 5–30 keV (detector energy threshold) and (iii) detected energy spectrum attenuated by the beam path in the energy range from 5–30 keV (see Section 3.2). The latter is calculated using the Lambert–Beer law for every simulated spectrum.

**Figure 8:**
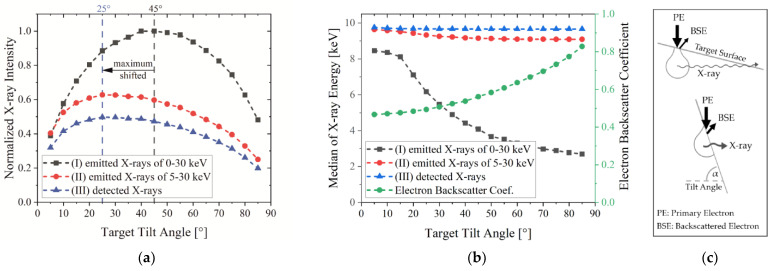
(**a**) Normalized X-ray intensity of a W bulk target depending on target tilt angle showing a shift in the maximum intensity to lower tilt angles due to PCXD energy threshold. Evaluated are different conditions: (I) emitted photons in range of 0–30 keV, (II) emitted photons in range of 5–30 keV and (III) detected photons; (**b**) median of X-ray intensity and BSC of W depending on target tilt angle; (**c**) scheme of interaction volume for low and high target tilt angles showing the number of backscattered electrons indicated by the arrow size and the distance that photons need to travel to leave the target.

As an example, Figure 8a shows the distribution of the W X-ray intensity depending on the target tilt angle for the three conditions. The intensity distribution is influenced by the electron backscatter coefficient (BSC), X-ray energy and photon absorption in the target itself, which is depicted in Figure 8b,c. To explain the behavior, one has to distinguish between low and high target tilt angles.

For condition (I), the X-ray intensity distribution depending on the target tilt angle is rather symmetrical with its maximum at an angle of around 45° (black graph). Using low and high target tilt angles leads to a strong intensity reduction.

The intensity using low target tilt angles is reduced, due to photon absorption in the target, as shown in Figure 8c. Photons need to travel long distances through the target material itself to reach the PCXD, which is positioned at a 90° angle to the incoming electron beam. Therefore, most low-energy photons are absorbed in the target before reaching the surface and the overall intensity is low. This effect can be proven by the evaluated median of the X-ray energy depending on the target tilt angle. As shown in Figure 8b, the X-ray energy decreases strongly for low target tilt angles, meaning that only high-energy photons are detected.

For high target tilt angles, the X-ray energy only slightly influences the behavior of condition (I) since high-energy and also low-energy photons can leave the target (Figure 8c). The reduction in the X-ray intensity at high target tilt angles is caused by the electron back scattering (BSC) shown in Figure 8b. The latter is constantly increasing with an increasing tilt angle to about 80%, so more and more electrons do not contribute to the X-ray emission. In summary, the target absorption and BSC result in a reduction in the X-ray intensity at low and high target tilt angles and lead to a maximum intensity at a tilt angle of around 45°.

Condition (II) reveals an overall strong reduction in the X-ray intensity since photons below the energy threshold of 5 keV including the characteristic line at 1.8 keV are eliminated from the spectrum. At target tilt angles smaller than 10°, most low-energy photons are absorbed within the target, leading to the similar values of conditions (I) and (II) in Figure 8a. This behavior differs for higher target tilt angles. As shown for condition (I), photons with an energy below 5 keV can leave the target for higher tilt angles but the detector threshold now eliminates these photons, so the X-ray intensity decreases significantly. Moreover, the BSC leads to a further reduction in the X-ray intensity, so the overall maximum of the X-ray intensity shifts from a target tilt angle of 45° to 25° and perfectly matches the experimental results presented in Figure 7.

In terms of the X-ray energy of condition (II), the 5 keV cut-off leads to a strong increase in the X-ray energy that barely changes for different tilt angles. This is a strong indicator that photon absorption in the target is less relevant for the overall X-ray intensity of condition (II).

As soon as the beam path of the XRM-II is taken into account, and only the detected photons are evaluated (condition (III)), the intensity is further reduced, but the W spectrum is rather weakly attenuated because of its relatively high X-ray energy.

**Figure 9:**
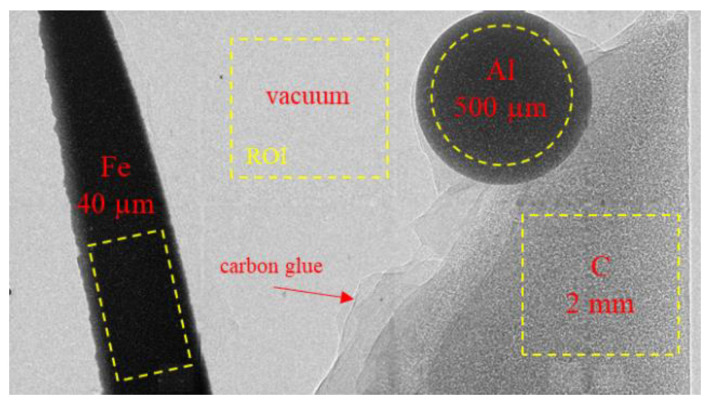
Radiographic image of a test object made of pure iron, aluminum and carbon to determine CNR. The marked areas in yellow are used for evaluation and the values correspond to element thickness. The aluminum ball is fixed to the carbon surface using carbon glue as indicated with the red arrow.

Based on Table 3, a target made of Cr and Mo represents materials with a low and high X-ray energy. In the following, we will focus on these two materials and also on Pt due to the high X-ray intensity and quantify the different image contrasts that can be reached. Therefore, a test object made of C, Al and Fe is placed into the beam path and images with different target materials are acquired. The exposure time is adjusted to an equal flatfield intensity for every target material. Figure 9 shows a radiography of the test object with different materials imaged and marked areas used to determine the CNR. To calculate the CNR, the method of Bechara [19,20] is applied and defined as:

CNR = contrast/noise = |µ_1_ − µ_2_|/sqrt (σ_1_^2^ + σ_2_^2^), (3)

µ is defined as the expectation value and σ as the standard deviation of the signal. All parameters can be obtained graphically from the image histogram. With the approximation of a Gaussian distribution of the signal, σ is given by:

FWHM ≈ 2.35 σ(4)

The resulting CNR is listed in Table 4. The use of different target materials leads to significant changes in the contrast. The target material Pt shows the highest contrast for imaging Al and Fe. These two materials represent light metals and period 4 elements, which are the predominant materials being investigated. Due to this performance, Pt is most suitable for applications in SEM-based nano-CT. A further useful target material is Cr since it delivers a high contrast for low-absorbing materials like polymers. The advantage of the high characteristic energy of Mo is not helpful in enhancing the image contrast and is unsuitable for most applications.

**Figure 10:**
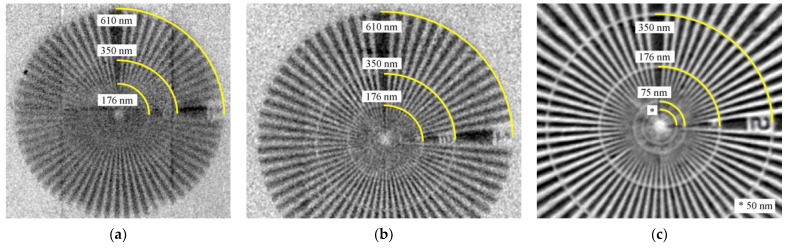
Radiographic images of a Siemens star acquired with a 25° tilted bulk target showing differences in spatial resolution as well as contrast (yellow arcs indicate the defined pattern size. The arc labeled with * corresponds to a pattern size of 50 nm): (**a**) image acquired with a pixel size of 76 nm using a Cr target; (**b**) image acquired with a pixel size of 32 nm using a Mo target; (**c**) image acquired with a pixel size of 45 nm using a Pt target.

To complete the characterization of different bulk targets, a Siemens star is imaged to obtain the spatial resolution. The electron beam current was set to 330 µA and the targets were tilted to around 25–30°. To avoid deviations in the target tilt angle between different target elements, all targets are glued on one tilted target holder. Nevertheless, this leads to a varying distance (micrometer range) between the target and Siemens star for the different target elements and results in different magnifications and pixel sizes. To prevent a collision of the target and Siemens star, the maximum magnification is limited. Images are acquired with a pixel size of 50 nm using a Cr target, 63 nm using a Mo target and 45 nm using a Pt target. All three targets were used to acquire 30 projections by integrating 24 images with an exposure time of 2500 ms. Post-processing was performed in ImageJ by applying a median filter with a radius of one pixel. After that, all 30 single images were summed up pixel per pixel using z-projection [21]. The images of the Siemens star are shown in Figure 10 where the yellow arcs represent the defined pattern size.

The target material Cr shows a clear anisotropy of the spatial resolution. On the one hand, an electron beam spot size of about 600 nm and a low scatter angle of electrons in Cr limit the horizontal extent of the X-ray focal spot, leading to a vertical spatial resolution of about 200 nm. On the other hand, the vertical extent of the X-ray focal spot is rather large due to the relatively high electron penetration depth in Cr, resulting in a maximum X-ray emission depth of about 3 µm, as shown in Table 2. Since characteristic X-rays determine about 80% of the entire X-ray spectrum (see Table 2) and their energy with 5.4 keV is rather low, photons used for imaging will be emitted from deep inside the target. This large vertical extent of the X-ray focal spot leads to a low horizontal spatial resolution of about 500 nm.

With the use of a Mo bulk target, the details of about 170 nm can be resolved since the electron penetration depth is smaller compared to Cr. Moreover, the entire X-ray spectrum contributes to X-ray imaging and not mainly low energy characteristic photons, leading to a reduction in the effective X-ray spot size. In addition, only a volume of high photon density determines the spatial resolution in X-ray imaging.

The target material Pt reveals a sharp and high contrast image due to the high X-ray intensity and narrow spatial X-ray distribution, leading to an image with less noise compared to Cr and Mo. Pt shows the lowest electron penetration depth for the investigated elements, limiting the X-ray focal spot so details in the size of about 125 nm can be resolved.

The 600 nm measured electron spot size and 1 µm maximum emission depth of 5 keV photons determined in Section 3.1.1 and Section 3.1.3 seem to be contradictory compared to the 125 nm spatial resolution in X-ray imaging but these values describe the X-ray focal spot size and not the effective X-ray focal spot size. The X-ray focal spot size is nearly equivalent to the interaction volume of the electron beam and target while the effective X-ray focal spot size is defined as the projection of the focal spot size onto the detector surface. The latter is significantly smaller and the only relevant parameter since it determines the spatial resolution in X-ray imaging. There are different facts underlining that the effective focal spot size is significantly smaller: the method to measure the electron spot size is an overestimation since it results in the maximum electron focal spot size regardless of the Gaussian electron beam distribution. Strongly scattered electrons lead to a large measured electron spot size even though they do not contribute to the effective X-ray focal spot. Moreover, the target tilt angle will lead to a lower electron penetration depth and 5 keV photons will only slightly contribute to X-ray imaging. The effective X-ray focal spot will be formed by photons with a higher energy. Furthermore, photon absorption due to the target tilt is not taken into account. The sum of these factors leads to a significant reduction in the effective X-ray focal spot size and to the measured spatial resolution of 125 nm. A quantification of the focal spot size of different target materials and its influence on the spatial resolution is planned for the future.

Using a Pt bulk target leads to the best performance by far regarding the X-ray intensity, spatial resolution as well as image contrast for most applications. Due to this fact, a Pt bulk target is recommended as a standard target with high photon flux. To image low-absorbing materials like polymers, a Cr bulk target will be useful to enhance the contrast. Negative consequences are a decreasing X-ray intensity and spatial resolution. To improve the spatial resolution, a Cr foil target will be prepared in the future. An additional diamond substrate will support heat flow to resist the high thermal electron beam input.

#### 3.3.2. Needle Target

**Figure 11:**
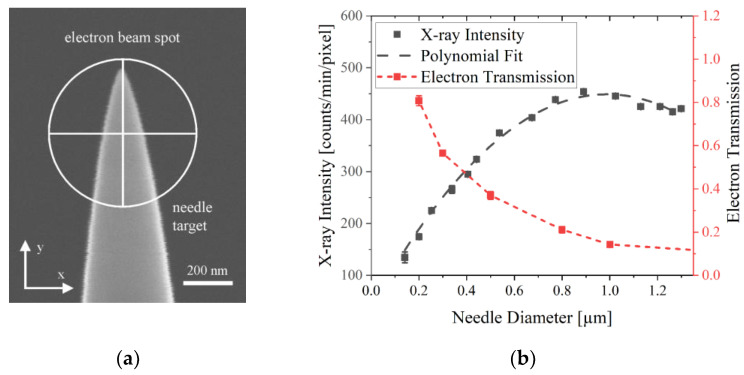
(**a**) SE image of a W needle target with a tip size of less than 100 nm and a scheme of electron beam spot size focused on a target; (**b**) detected X-ray intensity of a W needle target depending on electron beam positioning on different needle diameter.

The focal spot size of the X-ray source influences the spatial resolution in X-ray imaging systems based on geometrical magnification. Since the minimum electron beam spot size is about 600 nm (Figure 4), the X-ray focal spot can be further reduced by a smaller X-ray target. Therefore, we used commercially available W needles with a tip diameter of around 100 nm intended for SEM-based nano probing (Figure 11a). W is most suitable because of its high melting point of over 3400 °C and its high thermal stability. Materials with lower melting points will change shape due to the high thermal energy input of the electron beam and the tip radius will enlarge.

The graph in Figure 11b represents the X-ray intensity [counts/minute/pixel] depending on the needle diameter, which was acquired while the electron beam was positioned on the needle tip center. For every data point, the electron beam spot is moved in the needle tip direction (negative y-direction) by a certain distance, so the electron beam hits the target at a position equivalent to the displayed needle diameter. For larger needle diameters, the interaction volume increases and consequently, the X-ray intensity increases. Moreover, Figure 11b shows the amount of electrons which are collected underneath the needle target with a Faraday cup. It represents transmitted and primary electrons not interacting with the target. The graph clearly shows that the X-ray intensity will increase as long as the number of transmitted electrons decreases because of a larger interaction volume so more electrons contribute to the X-ray emission. In case the electron beam is positioned too far away from the tip, emitted X-rays are absorbed by the target itself and the intensity reduces.

As shown in Figure 11a, only a certain amount of primary electrons hit the needle target, limiting the efficiency of the X-ray excitation which illustrates a high importance in enhancing the electron beam density by a reduction in the WD. As a result, the spatial resolution is enhanced due to a smaller interaction volume and also the X-ray intensity is enhanced since more primary electrons hit the target. Moreover, a high number of transmitted electrons will lead to additional X-ray generation below the target (e.g., sample holder or sample itself), reducing the X-ray image quality.

The spatial resolution of a W needle target was already determined in previous works and reached 80 nm in 2D and 100 nm in 3D [2]. Consequently, a needle target made of W is most suitable for high-resolution imaging due to thermal stability and a small X-ray focal spot size.

## 4. Conclusions

In SEM-based CT, three different target geometries with varying target volumes are presented: bulk target, foil target and needle target. The geometrical limitation realized by reducing the target volume leads to a minimization of the X-ray focal spot, resulting in an enhanced spatial resolution in X-ray imaging but at the same time to a decreasing X-ray intensity. Furthermore, the electron beam itself has an influence on the X-ray properties, whereas an electron beam current of 300 nA or higher is suitable for SEM-based nano-CT. A lower current increases the image acquisition time tremendously and leads to inefficient measurements. Moreover, the distance between the electron focusing unit and X-ray target (working distance) should be as small as possible, as experiments show. As a result, the electron beam and X-ray focal spot size are reduced to a minimum, leading to an enhanced spatial resolution in X-ray imaging. In the case of a needle target, the X-ray intensity is increased because a reduction in the electron beam spot size is also equivalent to an increase in the electron beam density so more electrons hit the target. A method to determine the electron beam spot size using a high electron current (>300 nA) is also presented.

The X-ray intensity changes strongly between the emitted and detected spectrum due to the XRM-II setup. In addition to the absorption caused by a Be window, air and an Al layer, the X-ray detector is only sensitive for energies above 5 keV. Transferring the beam path to a vacuum will increase the X-ray intensity by about 20–50% depending on the target material. Consequently, the acquisition time for X-ray CT would decrease by about the same amount. The X-ray intensity of the bulk and foil target is strongly influenced by the target tilt angle. Experiments and simulations reveal that absorption caused by the setup leads to a maximum shift in the X-ray intensity from 45° to smaller target tilt angles of 25–30°.

For SEM-based CT, we recommend three targets for different applications:

- Platinum bulk target tilted by 25°: The target is a perfect combination of X-ray flux and spatial resolution. Due to the low penetration of electrons, the X-ray focal spot size is small and a 2D spatial resolution of about 125 nm is reached. At the same time, a combination of 30 keV excitation energy and 9.4 keV characteristic X-ray line energy leads to the highest X-ray intensity. Due to these properties, a Pt target is suitable as a standard target for SEM-based CT. It is especially recommended for imaging high-absorbing or large samples and for fast CT scans.
- Chromium bulk or foil target tilted by 30°: Imaging low-absorbing materials like polymers, target materials with their characteristic line energy right above the energy threshold of the detector are recommended. Following these recommendations, Cr is chosen for the presented system. In addition to an enhanced image contrast, a lower X-ray intensity and spatial resolution have to be expected as disadvantages due to the low probability of X-ray excitation and high electron penetration depth. The spatial resolution will be enhanced by changing the geometry from bulk to foil, but only with a further loss of the X-ray flux.
- Tungsten needle target: To reach the highest spatial resolution and reveal small details in a specimen, a needle target with a tip diameter of about 100 nm made of W is recommended. The geometry limits the X-ray focal spot size, and consequently, enhances the spatial resolution up to 80 nm (2D), while still delivering enough photons for imaging. Moreover, W is able to resist the high thermal energy input of the electron beam due to its high melting point. The spatial resolution and X-ray intensity can be adapted in a specific range according to the requirements of the measurement by changing the electron beam position onto the target tip.

## Figures and Tables

**Table 1:** Diameters of objective lens apertures and NC.

OLAP	NC	A0	A1	A2	A3	A4
Diameter [µm]	2000	1000	110	70	50	30

**Table 2:** Target materials, their melting point, relevant characteristic X-ray energy and the amount of characteristic photons excited with 30 keV electrons regarding the entire X-ray spectrum in the energy range between 5 and 30 keV.

Element	Melting Point [°C]	Char. X-ray Line Energy [keV]	Amount of Char. X-ray Photons [%]	Max. Emission Depth of 5 keV Photon [µm]
Cr	1857	Kα = 5.4	79	3.1
Fe	1538	Kα = 6.4	73	2.8
Cu	1083	Kα = 8.0	62	2.5
Y	1523	Kα = 15.0	18	5.2
Mo	2617	Kα = 17.5	9	2.6
W	3410	Lα = 8.4	35	1.3
Pt	1772	Lα = 9.4	31	1.2

**Table 3:** All values relate to an energy range of 5–30 keV. The table shows X-ray intensity of simulated X-ray emission and calculated detection normalized to the emitted intensity of Cr. In addition, absolute transmission and median of detected X-ray energy are listed.

Element	Normalized Emitted Intensity	Transmission of Setup [%]	Normalized Detected Intensity	Median of Detected X-ray Energy
Cr	1.00	41.0	0.41	5.5
Fe	0.87	56.5	0.49	6.4
Cu	0.70	71.5	0.50	8.1
Y	0.40	77.5	0.31	13.4
Mo	0.37	78.5	0.29	12.3
W	0.78	75.5	0.59	8.7
Pt	0.73	78.0	0.57	10.9

**Table 4:** Calculated CNR for different materials and targets. Exposure time was varied to reach a similar mean flat-field intensity.

Target Material	Exposer Time [min]	Flat-Field Intensity	CNR for Imaging		
			C	Al	Fe
Cr	8	1940 ± 20	2.8	8.4	14.2
Mo	5	2020 ± 60	1.5	8.3	15.2
Pt	3	2180 ± 70	1.8	12.8	20.2

## Data Availability

Data is contained within the article.
